# Journal of Global Health's GUidelines for Authors on Requesting and DIsclosing changes in Authorship Nominations (GUARDIAN)

**DOI:** 10.7189/jogh.16.01003

**Published:** 2026-04-15

**Authors:** Luka Ursić, Harry Campbell, Igor Rudan

**Affiliations:** 1 Department of Research in Biomedicine and Health, Center for Evidence-based Medicine, University of Split School of Medicine, Split, Croatia; 2 Empirica, Split, Croatia; 3 Centre for Global Health, Usher Institute, The University of Edinburgh, Edinburgh, UK; 4 Nuffield Department of Primary Care Health Sciences and Green Templeton College, Oxford University, Oxford, UK

## Abstract

We address the growing concern of requests for post-submission and post-acceptance changes in authorship which our editorial team has observed in recent years. We emphasise that authorship order should be agreed upon by all contributors before submission, as all authors are expected to approve the final version of their manuscript and agree on their respective authorship positions. This editorial identifies seven categories of authorship change requests, ranging from adding or removing ordinary authors to modifying first or last authorship positions, or introducing group authorship. We consider some of these requests legitimate, such as adding author(s) who performed additional analyses based on reviewers’ feedback or removing author(s) disagreeing with such revisions. We consider some others indicative of potentially concerning practices, particularly those involving changes to first or last authorship positions. We place this trend within a broader context of questionable research practices, including ‘honorary’ or ‘gift’ authorships driven by institutional power dynamics and the more recent emergence of ‘paper mills’. These practices seem to be increasing in frequency with the rise of artificial intelligence (AI) and large publicly available data sets, which have lowered the barriers to producing large volumes of research of questionable value. Existing safeguards developed by organisations such as the International Committee of Medical Journal Editors (ICMJE) and the Committee on Publication Ethics (COPE) are helpful, but limited in their ability to prevent such practices. To address these challenges, the *Journal of Global Health* introduces the GUidelines for Authors on Requesting and DIsclosing changes in Authorship Nominations (GUARDIAN), which mandate full transparency whenever authorship changes occur after submission. Specifically, in the part of the standard ‘acknowledgements’ section at the end of each paper, where the mandatory ‘authorship contributions’ statement is typically detailed, the authors will be required to: (i) declare that the authorship byline has been changed since submission; (ii) disclose precisely what changes to the byline occurred between these two versions; (iii) provide an explanation for this change; and (iv) provide the final authorship contributions accordingly. Supporting documentation will also be archived by our editors and may be shared upon legitimate requests. The GUARDIAN aim to deter misconduct through transparency, protect early-career researchers from authorship pressure, and improve accountability in academic publishing. Together with our previously introduced Guidelines for Reporting Analyses of Big Data Repositories Open to the Public (GRABDROP) and other integrity initiatives, the GUARDIAN represent a proactive effort to safeguard credibility of authorships, while allowing legitimate adjustments whenever they are properly justified.

In recent years, the editorial team of the *Journal of Global Health* (*JoGH*) has noted an increased frequency in requests for modifying the authorship byline in research papers, either during peer review or, more frequently, at the post-acceptance stage (**Box 1**). This trend is concerning, especially as all authors are expected to read and approve the final version of the paper and agree with their position in the byline before they submit it to *JoGH*. Clearly, such pre-submission check needs to also include the understanding their position in the order of authorships and accepting it. Therefore, we expect from the authors to resolve all issues pertaining to the authorship order *before* they submit their articles to our journal. However, recognising that post-submission changes are, in some cases, still valid, organisations such as the International Committee of Medical Journal Editors (ICMJE) and the Committee on Publication Ethics (COPE) provide criteria and guidance on how these cases should be handled [1,2]. For example, in cases when authors are added to a paper post-acceptance, COPE suggests editors request from the authors a reasonable explanation, a new authorship declaration, and a signed form from all co-authors confirming that the new contributors fulfil the journal’s authorship criteria [3]. In this editorial, we introduce our journal’s GUidelines for Authors on Requesting and DIsclosing changes in Authorship Nominations (GUARDIAN), which aim to supplement current ICMJE and COPE guidelines in view of two specific challenges against which they have been, in our experience, largely ineffective – ‘honorary’ or ‘gift’ authorships and ‘paper mills’.

Box 1 **Types of requests for authorship changes experienced by the JoGH editorial team**

We have been witnessing changes to authorship nomination practices over the years: increasingly complex papers are being developed through the collaboration of many scientists, leading to a growth in the number of articles with many co-authors. In some cases, there is a need to share both the first and the last authorship – a practice we accepted as a logical consequence of scientific progress. We do not normally restrict the number of possible joint first and joint last authors, although we expect it to be reasonable and correspond to the complexity of the underlying research work. As a benchmark for authors, we would be surprised if there are more than three authors deserving of either joint first or joint last authorship, although this can be allowed in some cases, such as where a genuinely different and highly complex paper is developed by many collaborating research groups.

Besides the introduction of joint first and joint last authorships, we began encountering requests for authorship changes either during the process of the revision of a paper, or after its acceptance and before its publication. These requests can theoretically fall in several different categories:

- requests to add one or more new co-authors as the (joint) first, (joint) last, and/or corresponding authors;
- requests to ‘upgrade’ one or more of the listed ‘ordinary’ co-authors to (joint) first, (joint) last, and/or corresponding authors;
- requests to add one or more new co-authors in the position of ‘ordinary’ co-authors, *i.e.* to positions other than those of (joint) first, (joint) last, or corresponding authors;
- requests to remove one or more (joint) first, (joint) last, and/or corresponding authors;
- requests to ‘downgrade’ one or more of the listed (joint) first, or (joint) last, and/or corresponding authors to ‘ordinary’ authors;
- requests to remove one or more ‘ordinary’ co-authors from the authorship list;
- requests to add the group authorship in addition to the list of authors.

## REQUESTS FOR AUTHORSHIP CHANGES AS A CONSEQUENCE OF POWER RELATIONS AND PAPER MILLS

Authorship manipulations have pervaded science long before the emergence of paper mills. Honorary or gift authorships, assigned to individuals based on power relations within the institutions to which the authors are affiliated (such as their seniority, career advancement needs, or other reasons) rather than their contribution, have been identified as a prevalent questionable research practice (QRP) [4–7].

Besides this long-standing issue, the scientific community has recently begun seeing another reason to be wary regarding the veracity of the contributions of (co-)authors listed on research papers – the emergence of the so-called paper mills. These entities comprise individuals, groups, or organisations that mass-produce low-quality or entirely fabricated research papers and sell their authorship slots to individuals who wish to use them in advancing their careers [8,9]. These papers are then assessed by journal editors and proceed to one of five ‘pathways’ [9,10]:

1. they are considered to be unworthy of publication and are rejected (majority of cases);
2. they are sent out for review and eventually accepted for publication, in part due to an insufficiently detailed and/or critical peer review;
3. they are forwarded to author-suggested peer reviewers, who may be paper mill members hidden behind fictitious e-mail addresses; they tend to provide favourable reviews to the unaware editor, who then accepts such papers for publication;
4. they are processed by editors who can be prompted, influenced, or incentivised by paper mills to conduct a less rigorous peer review and accept the paper for publication;
5. they are submitted to predatory journals that were set up as a part of the paper mills cycle, with questionable editors and peer reviewers, who then accept it for publication.

Only the first scenario protects the scientific community from the flood of papers produced by paper mills. The second and the third scenarios are concerning because they result in papers slipping through ‘editorial gatekeeping’ without anyone’s real fault or malicious intent. In contrast, the last two scenarios clearly constitute research misconduct.

At the *JoGH*, we oppose practices that lead to misrepresentation of true authorships and do not follow existing ICMJE and COPE authorship guidelines [1,2]. We also understand that this scientific malpractice is driven by competitive academic cultures that incentivise researchers to publish many papers quickly and thus advance their careers. It is, therefore, unsurprising that some of these paper mills proliferate through a network of authors, brokers, and even journal editors [11].

Historically, the growth of paper mills has been limited by the challenge of producing research papers, even if they were of low-quality or fabricated. However, the emergence of digitalisation and artificial intelligence (AI) based tools, particularly large language models, coupled with the rise of ‘open science’ and the availability of large-scale, publicly accessible data sets [12,13] created the circumstances that led to a massive influx of rapidly generated papers [14]. Acknowledging the negative impact of paper mills and their products on the integrity of science and the reliability of our knowledge base [15], stakeholders within the publishing industry have joined efforts in the United2Act initiative [16]. Their aim is to identify the key characteristics of paper mills and their operations, to understand why researchers use them, and to educate researchers better on the negative impact of such papers on science [16].

## LIMITATIONS IN THE CURRENT ICMJE AND COPE GUIDELINES IN TACKLING THE ISSUES

While exceptionally useful in defining and explaining (in)appropriate practices, current ICMJE and COPE guidelines and flowcharts are of limited use for practically addressing these authorship malpractices – *i.e.* power-based honorary and gift authorships, and authorships in paper mills. As an example, all the authors of such questionable papers have an incentive to provide the signed authorship form and a new declaration when adding a new contributor, especially if the individual agreed to participate in covering the article processing charge (APC) – because their joint interest is to eventually publish the paper. A cautious editor could then ask for data sets, analytical code, or other proof of the new co-author’s contribution. If these authors fail to produce these materials, or if the ones they do produce do not seem genuine, this should then raise major concerns.

However, this gatekeeping activity is far more complex in practice. Reanalyses of large-scale, publicly available data sets, for example, have been notoriously difficult to handle by the editors [13], as they can be fully conducted and reported using AI-based tools. Other evidence, such as dated e-mail communication or manuscripts with tracked changes, could also be requested, but could still theoretically be falsified. Given that the editor should presume all authors innocent of any research misconduct until proving otherwise, and given the burden of time and the difficulty of proving misconduct in such complex papers, the editors are incentivised towards judging an author’s contribution to be legitimate and allowing their addition to the byline.

## OUR POSITION ON THE DIFFERENT TYPES OF REQUESTS FOR AUTHORSHIP CHANGE

**Figure 1:**
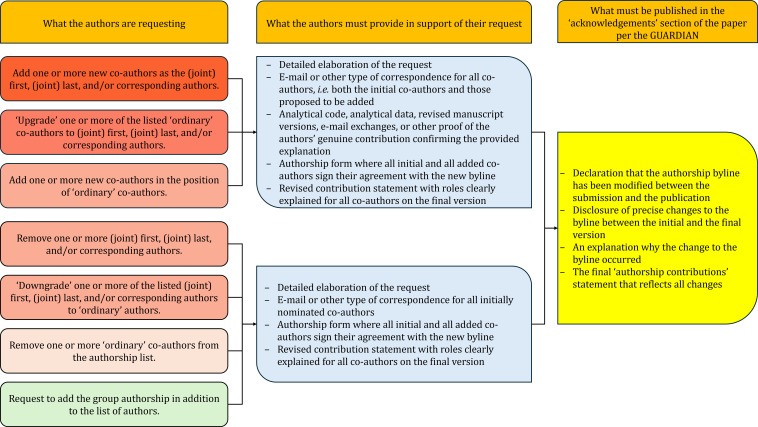
Flowchart showing how requests for changes should be handled per the GUARDIAN. Darker shades of red indicate cases where questionable activity or misconduct are more likely, and that will prompt deeper investigation, while lighter shades of red and green indicate requests that are less concerning. This ordering/grouping is primarily based on the experience of our editorial team with authorship change requests in the past.

We have already explained seven theoretically possible attempts to change authorships post-submission (**Box 1**). Now, we propose appropriate responses to each of these cases through a GUARDIAN-based flowchart (**Figure 1**), which will alleviate the pressure for editors and gradually help expose all those who serially engage in authorship-related QRPs.

Cases where the authors request the addition of a new (joint) first/last or corresponding author will, per the GUARDIAN, be treated with extreme caution, as they might be indicative of authorship-related QRPs or paper mill activity [4–10]. We believe it is highly unlikely that an author contributed sufficiently to earn the most ‘prestigious’ position in the byline post-submission. Unless there is a strong case that either an existing ordinary co-author or an entirely new co-author made such an important contribution during the review process that they truly deserve a joint-first or joint-last authorship – which we do not think is a common scenario –we will consider this as a signal of a potentially concerning authorship practice and will generally decline such requests. However, we do recognise very rare legitimate cases of such requests, where existing ordinary co-authors might know how to conduct the requested additional analyses, so they might then be upgraded to a joint first or last author by consensus, or where a senior ordinary author could bring in some additional funding needed to acquire analytical software and ‘earn’ joint senior authorship. However, all such legitimate cases of authorship change need to be well-documented to the editors. Simultaneously, we recognise that the addition of ordinary co-authors during or after the peer review/revision process can also be legitimate. We have had cases where reviewers requested additional analyses to be performed which were outside of the original team’s capacity, prompting the invitation of a new author to address these comments.

Cases where the initial (joint) first or senior authorship authors are either removed from the byline entirely or downgraded to ordinary authors, or where ordinary authors are removed from the paper are, in our experience, rare. They might occur legitimately, *e.g.* if the author in question disagrees with peer reviewers’ comments and the subsequent changes to the paper, so they wish to be removed from the byline. These cases can also result from disagreements between authors during the process of getting the paper published. In all such cases, we will request a form signed by all initial co-authors, including those who are downgraded or removed, and a revised contribution statement that reflects this change. The most concerning, but quite possible scenario, is that a downgrade or removal of an author is requested as a consequence of power relations. So, even with all the signatures received, we will still reach out to the downgraded or removed co-authors separately to confirm their consent.

Occasionally, authors may ask to add the name or acronym of their research collaboration to the end of their byline. We usually agree to such requests, as they are mainly put forth by collaborations whose record can be tracked easily in bibliographic databases, and as the group name is typically added at the end of the list of authors. Some journals request that all the additional names of co-authors who are the members of that group, but not already listed as co-authors in the paper, are displayed in the acknowledgement or in the supplementary online material. We do not request this, as we recognise the need of some research groups to ‘brand’ their papers with the name of their collaboration so that they could be searched for more easily in the databases of scientific articles. However, if the authors also request the acknowledgement of some additional co-authors under a group name, we normally allow this.

As we cannot *a priori* predict the nature of these requests, we will ask authors requesting the addition or upgrading of (joint) last, (joint) senior, (joint) corresponding, or ordinary co-authors for signed authorship change forms which will be accompanied with a detailed elaboration of their requests and a revised contribution statement. To support their request, they will have to provide e-mail or other type of correspondence with the author being added or upgraded, as well as analytical code, analytical data, revised manuscript versions, or other proof of their contribution that reflects the provided explanation. This supporting documentation will be archived by our editors and may be shared upon a legitimate request.

## HOW OUR NEW GUIDELINES WILL HELP WITH HANDLING AUTHORSHIP-RELATED MATTERS

As seen from the GUARDIAN-based flowchart (**Figure 1**), all these cases will, at the end, be publicly disclosed in the part of the standard ‘acknowledgements’ section at the end of each paper, where the mandatory ‘authorship contributions’ statement is typically detailed. There, the authors will be required to:

1. declare that the authorship byline has been modified between submission and publication;
2. disclose precisely what changes to the byline occurred between these two versions;
3. provide an explanation why this change occurred;
4. provide the final ‘authorship contributions’ statement that reflect this new change.

We expect that the adoption of the GUARDIAN into our workflow will lead to three positive effects. First, this will disincentivise any authorship-related misconduct which is typically driven by a want for career progress. In doing so, it will ‘protect’ early career researchers’ position on the paper, whenever they are pressured by their seniors or their peers for authorships that have not been fully earned, or when they are forcibly demoted to lower positions through the same power relations.

Second, this transparency will allow members of the research community who know the authors and their work, especially those who eventually decide on the further career progress based on all such papers, to judge how realistic the authorship change and contributions statements are and to assess the validity of their explanation of this change. Any evidence related to the authors’ request will be archived by *JoGH*’s editorial team, who reserve the rights to share it with relevant institutions upon request.

Third, we believe this will also disincentivise any submissions originating from paper mills, which will also alleviate our editorial team’s workflow. We have already begun addressing the evolving challenge of paper mills by developing the Guidelines for Reporting Analyses of Big Data Repositories Open to the Public (GRABDROP), which aim to limit the impact of paper mill-produced secondary data analyses, while still acknowledging legitimate research efforts, especially from under-funded and under-represented settings [17]. While they have already received some recognition in the wider research community [18–20], the GRABDROP and the associated checklist mainly prove useful at the stage of editorial evaluation. However, a gap remained in dealing with requests for authorship changes that come up between a paper’s submission and its eventual publication. The GUARDIAN addresses this directly by requiring full transparency with all post-submission authorship changes, meaning that any journals adopting this approach are less likely to be targeted by paper mills that base their work on post-submission authorship changes [8–10].

The integration of our two new guidelines (GRABDROP [17] and GUARDIAN) and our planned adoption of the STM Integrity Hub [21] into our editorial processes should offer strong protection against authorship-related QRPs and paper mills, while still enabling the evaluation and acceptance of legitimate pre-publication authorship changes and appropriate analyses of publicly available big data repositories. We will monitor their effectiveness and look for any shifts to submission patterns and requests for authorship changes, so that we can inform and improve our editorial policies and processes. We will also welcome comments and suggestions for improvements and updates to these new guidelines, wherever appropriate.
